# Timely monitoring of total mortality associated with COVID-19: informing public health and the public

**DOI:** 10.2807/1560-7917.ES.2020.25.34.2001591

**Published:** 2020-08-27

**Authors:** Lasse S Vestergaard, Kåre Mølbak

**Affiliations:** 1 Statens Serum Institut, Copenhagen, Denmark

**Keywords:** COVID-19, pandemic, mortality monitoring, public health action

Following its emergence at the end of 2019, the novel severe acute respiratory syndrome coronavirus 2 (SARS-CoV-2) and the associated coronavirus disease 2019 (COVID-19) rapidly developed into a global pandemic, causing millions of cases and hundreds of thousands of deaths in Europe and worldwide over the past months [1-3]. This event is a stark reminder of the need to stay alert and be prepared to handle new and unexpected public health threats at all times. The COVID-19 pandemic has demonstrated once again that timely and systematic epidemiological monitoring of disease signals is an essential part of public health preparedness and action that guides prevention and control strategies and informs policymakers and the public. Such monitoring requires constant attention and effort, careful coordination and ongoing support to deliver timely, objective and unbiased information.

Mortality is a key signal of disease severity. However, measuring mortality caused by an unknown disease is challenging in the absence of established testing methods, and may be further impacted by inadequate health systems capacity to handle testing and affected patients. Even though COVID-19 testing was quickly established and rolled-out in many settings, a considerable number of COVID-19 patients might have never reached the point of testing for a wide range of logistical and other reasons. These cases constitute a considerable number of undetected and unreported COVID-19 deaths, while the official mortality statistics only paint a partial picture. In this situation, monitoring of excess all-cause mortality has become widely recognised as providing an independent and unbiased measure of COVID-19-associated mortality. Indeed, weekly excess mortality monitoring has already contributed importantly to our understanding of whom, where and when the COVID-19 pandemic has affected the most as it has evolved.

Over the past weeks, *Eurosurveillance* has published a series of Rapid communications reporting estimates of the excess mortality attributable to COVID-19 during its first wave in Europe [4-7]. Together, they paint a grim picture of the severity of COVID-19, documenting the unprecedented steep mortality peak in Europe within weeks, and how it has affected particular age groups and particular geographical areas. The papers also illustrate how effective the public health responses have been in controlling the pandemic and reducing population mortality. They build on the same well-established methodological approach, applied every week by the 24 countries or sub-national areas participating in the European monitoring of excess mortality for public health action (EuroMOMO) network (www.euromomo.eu). EuroMOMO is a European mortality monitoring activity that aims to detect and measure excess deaths related to seasonal influenza, pandemics and other public health threats. Official national mortality data are provided weekly from each of the participating European countries to the network hub, supported by the European Centre for Disease Prevention and Control (ECDC) and the World Health Organization (WHO) Regional Office for Europe, and hosted by the Statens Serum Institut, Copenhagen, Denmark. Set up back in 2009 in response to the 2009-influenza A (H1N1) pandemic, EuroMOMO has since, without interruption, produced its weekly mortality outputs, informing national and international public health and disease agencies, and the public. The mortality outputs are routinely used to assess the severity of the annual influenza season during the European winter and the impact of local heatwaves during the summer. But as mentioned, EuroMOMO was also established as a means to determine the impact of new pandemics or other yet unknown public health threats.

The quantitative estimation of excess all-cause mortality is done by comparing the observed number of deaths with the expected number of deaths (baseline). The weekly expected number of deaths is derived using a statistical model developed by EuroMOMO, i.e. a time-series Poisson regression model to predict the baseline, adjusted for a linear or nonlinear trend and seasonal variation [8]. The excess deaths are expressed either as numbers, or as proportions of the expected number of deaths, or using a standardised indicator around the expected number of deaths (z-score). This enables the comparison of mortality variations over time, as well as of age groups, sex and different geographical areas.

During the COVID-19 pandemic, the monitoring work of the network has shown its value in pioneering the demonstration of the evolving mortality impact of COVID-19 at population level. It has shown how the pandemic has a real impact on all-cause mortality, which is unlikely to be limited merely to a change in the spectrum of the causes of deaths or earlier deaths of frail and elderly people. As shown in each of the published national reports from Italy, England and France [5-7], and in the pooled EuroMOMO analysis [4], COVID-19 hit Europe in a first wave in early March 2020 and peaked within a few weeks. Hereafter, it declined rapidly following the implementation of comprehensive control and prevention interventions, which included physical distancing, attention to hand hygiene and in some countries, complete society lockdowns. However, the mortality impact varied from country to country. A systematic analysis of the mortality impact and how it relates to control measures is yet to come and will probably have to await the end of the year as we still do not know what will happen in the coming autumn and winter. However, preliminary data indicate that early implementation of physical distancing measures had a beneficial impact on mortality [9].

Although all papers published in *Eurosurveillance* demonstrate that the oldest age groups were by far the worst affected, they also showed that the middle-aged and even the younger age groups 15 to 44 years of age experienced excess mortality. Further, in Italy, COVID-19- associated mortality was found to be higher in men than in women [5]. Data from France confirm this observation [7]. Another key observation in the national excess mortality reports from Italy, England and France is the striking variation in mortality observed between different geographical areas, some even located close to each other [5-7]. Hence, the geographic heterogeneity seen across European countries is also reflected at the national level. This can probably be ascribed to the general heterogeneity of SARS-CoV-2 transmission, which is different from the generally more homogenous transmission of, for example, influenza viruses [10,11]. Another contributing fact was that national lockdowns resulted in a frozen epidemic, for example transmission was intense in northern Italy while at the same time it was limited in central and southern Italy, which is now mirrored in national mortality statistics [12]. As the pooled EuroMOMO analyses do not include mortality data on sex and sub-national differences, these national excess mortality reports provide additional key information to guide national COVID-19 response plans.

Outside Europe, the estimation of excess all-cause deaths has also provided an important picture of the overall mortality of the pandemic. Using slightly different but similar methodological approaches, a retrospective analysis from the United States demonstrated the grave overall mortality during the first wave of COVID-19 and the marked variation between areas and states [13]. A separate analysis demonstrated how excess all-cause mortality compares to mortality caused by other diseases such as heart disease, diabetes, cerebrovascular disease and Alzheimer disease, which may all have contributed to COVID-19 fatalities [14].

It is worth mentioning that in Europe, the first wave of COVID-19 occurred during the spring, thus at a time when there was not much mortality attributable to winter-influenza or summer-heat waves. This has allowed a more distinctive estimation of the excess mortality attributable to COVID-19. However, as we enter into the coming autumn and winter season, the weekly estimation of COVID-19 mortality and hence our means of monitoring the evolving epidemic will be more challenging. Here we have highlighted the marked heterogeneity, both within and between countries. It is uncertain whether areas that were relatively spared in the spring are at a higher risk from COVID-19-related mortality during the coming autumn and awareness is warranted. There will be a further need to develop new statistical models that can disentangle the relative contribution of COVID-19, influenza and infections with other respiratory pathogens to excess population mortality. Important lessons may be learned from the use of the FluMOMO model, which has been used to estimate influenza-specific mortality based on all-cause mortality and reported influenza activity [15,16]. Concerted efforts will be needed to quickly develop and set up similar new monitoring systems.

Indeed, numerous questions remain to be answered to fully understand the epidemiology and impact of COVID-19. So far, systematic weekly and retrospective all-cause mortality monitoring has told us that the total mortality of COVID-19 is considerably higher than judged from the official COVID-19 mortality statistics, and that younger adults and middle-aged individuals are not spared. In areas without reliable routine disease surveillance and mortality reporting systems in place, the collection and analysis of simple excess all-cause mortality data may provide some particularly valuable information to guide local public health action and to inform the public. Indeed, during the first wave of the COVID-19 pandemic in Europe, the EuroMOMO network received much interest from the public and media worldwide, keen to understand the true mortality associated with COVID-19.

To support the expansion of timely monitoring of all-cause mortality beyond Europe, the WHO has recently launched a new surveillance initiative, aiming to collect weekly mortality data from as many countries worldwide as possible [17]. This commendable initiative should be fully supported by all concerned parties, and coordination and synergies should be promoted for optimal and timely collection of national-level data and analysis, as well as ensuring public dissemination. Collaborative efforts are required to bring together and consolidate any new information on the COVID-19 pandemic as it becomes available, to guide effective mitigation efforts and other relevant public health action worldwide.
